# A prognostic insight of the mRNA vaccine against antibiotic-resistant bacteria

**DOI:** 10.1097/MS9.0000000000001970

**Published:** 2024-04-10

**Authors:** Mai Abdel Haleem Abusalah, Priyanka Choudhary, Hichem Bargui, Naveed Ahmed, Manal Abdel Haleem Abusalah, Om Prakash Choudhary

**Affiliations:** a Department of Medical Laboratory Sciences, Faculty of Allied Medical Sciences, Al-Ahliyya Amman University, Amman, Jordan; b Department of Veterinary Microbiology, College of Veterinary Science, Guru Angad Dev Veterinary and Animal Sciences University (GADVASU), Rampura Phul, Bathinda, Punjab, India; c Faculty of Pharmacy of Monastir, university of Monastir, Tunisia; d Department of Medical Microbiology and Parasitology, School of Medical Sciences, Universiti Sains Malaysia, Kubang Kerian, Kelantan, Malaysia; e Department of Veterinary Anatomy, College of Veterinary Science, Guru Angad Dev Veterinary and Animal Sciences University (GADVASU), Rampura Phul, Bathinda, Punjab, India

Highlights

- The antimicrobial resistant^1^ bacteria are highly infectious and increases the incidence rates of mortality and morbidity.
- The Antimicrobial resistance bacteria has a significant economic impact in terms of long-term hospital stays and treatment expenses, putting an enormous impact on healthcare systems.
- The increasing incidence of Antimicrobial resistance poses a challenge to contemporary medicine.
- Inventive approaches to managing antibiotic-resistant bacteria, including the generation of novel medications, are urgently needed.
- The mRNA therapy is considered as a potential treatment, where this therapy can transform the approach of vaccination, particularly against multi-drug-resistant (MDR) pathogens, cancer immunotherapy, protein replacement therapy, and other areas of contemporary medicine.

The WHO has highlighted caution once again about the inadequate number of novel antibacterial therapies being developed to combat the growing problem of antimicrobial resistance^1,2,3^. Antibiotic resistance develops when previously effective treatments are unable to control or kill bacteria via several methods including (i) drug inactivation by structural alteration or hydrolysis, (ii) target access prevention via reduced permeability of the membrane or upregulation of efflux pumps, (iii) modification of antibiotic targets via post-translational modifications or mutation^4,5^. Therefore, drug-resistant bacteria are the result of a complex evolutionary process influenced by a range of factors. These include selective pressure from antibiotic use, horizontal gene transfer, mutational adaptation, drug inactivation (often facilitated by an enzyme-catalyzed irreversible cleavage), modification of the antibiotic binding site, diminished drug accumulation (either through increased efflux or decreased membrane permeability), and ribosomal gene transfer^6,7^. Antibiotic misuse in both animals and humans, frequently with minimal or no therapeutic value, is a major contributor to resistance^4^. It was hypothesized that the significant role in the spread of antibiotic resistance is the pressure placed by antibiotic treatments on bacteria other than the targeted pathogenic organisms (“bystander selection”)^8,9^. The mobile resistance genes such as New Delhi metallo-β-lactamase 1 (NDM-1) and mcr-1 are quickly propagating around the world and causing resistance to a wide range of antibiotics, such as cephalosporins, penicillin, and carbapenems^10^. The most prevalent NDM-1-producing bacteria are *Acinetobacter* spp., *Enterobacteriaceae* (*E. coli*, *P. mirabilis* and *K. pneumoniae*), and *Pseudomonas aeruginosa*, where colistin is considered as the sole effective potential antibiotic. Nonetheless, the increase of the mcr resistance gene (mcr-1) is resulting in the emergence of pan-resistant strains^11^. This is most likely related to the widespread usage of colistin to boost pig growth in Chinese farms. The mcr-1 has also been detected in specimens from infected individuals in addition to raw meat^11^. As a result, the increasing number of multi-drug-resistant (MDR) pathogens, which are highly infectious and cause high incidence of mortality and morbidity, has a significant economic impact in terms of long-term hospital stays and treatment expenses, putting an enormous impact on healthcare systems. Furthermore, the increasing incidence of Antimicrobial resistance (AMR) poses a challenge to contemporary medicine, and inventive approaches to managing antibiotic-resistant bacteria, including the generation of novel medications, are vitally needed^10^.

Only 12 antibiotics have been approved since 2017, with ten of them belonging to existing classes with well-established AMR mechanisms^2^. A group of pathogens with the greatest mortality risk, including *Acinetobacter baumannii*, E*nterococcus faecium*, *Enterobacter*, *Klebsiella pneumonia*, *Staphylococcus aureus* and *Pseudomonas aeruginosa* were designated with the acronym ESKAPE^12,13^. The WHO has identified ESKAPE bacteria and multi-drug-resistant Mycobacterium tuberculosis (MDR-TB) as two of the twenty bacteria that require novel antimicrobials immediately, where it designates three distinct categories: critical, high, and medium priority^14^. The most concerning antibiotic-resistant bacteria, in addition to MDR-TB, are carbapenem-resistant (CRE) and extended-spectrum β-lactamases (ESBLs) Enterobacteriaceae (such as Carbapenem-resistant *P. aeruginosa* and *A. baumannii* and extended-spectrum β-lactamases (ESBLs) or carbapenem-resistant *K. pneumoniae*, which are classified as a group of critical priority pathogens) and methicillin-resistant *Staphylococcus aureus* (MRSA), which is classified as the high-priority group. Lastly, the medium-priority category comprises *Haemophilus influenzae*, *Streptococcus pneumoniae*, and *Shigella* spp^14^. The WHO 2021 pipeline report, issued in May based on 2020 data indicates that a total of 27 novel medicines are in clinical development against priority pathogenic organisms such as *Pseudomonas, Clostridioides difficile, Mycobacterium tuberculosis, Acinetobacter*, and various *Enterobacteriaceae* (including *Serratia*, *Klebsiella*, *Proteus* and *E. coli*), *salmonella* and *gonorrhoea*, where four were developed in 2017^2^. Furthermore, this report outlines some of the challenges to drug development, including the lengthy approval process, and poor rate of success^15^. Empiric therapy is the sole option because rapid identification of the exact infectious organism at the point of care is the major challenge. In reality, to manage all of the major microorganisms responsible for a specific infection, empiric therapy necessitates the use of broader spectrum treatments^10,16^.

Vaccines might become an essential and effective tool in combating AMR^17^. Importantly, the role of resistance mechanisms is reduced in the context of vaccination. Vaccines, unlike antibiotics, are intended to prevent illness^1,18^. Their usage is employed as a preventative measure, the host can begin developing an immune response before coming into contact with the pathogen, or even when there are only a few hundred or a few thousand bacteria present in the early stages of infection^19^. Vaccination reduces the probability of the emergence of resistance mechanisms, which can develop stochastically among billions of bacteria. In addition, vaccinations induce T cell or/and host-specific antibody responses, whereas most antibiotics only affect one target. Several mutations may be required to generate resistance to vaccines even, thereby complicating the establishment of microbial resistance^19^. According to the WHO, vaccinations protect between 2 and 3 million individuals worldwide each year from diseases including tetanus, whooping cough, influenza, and measles^20^.

Although vaccination is the best course of action to address AMR in many aspects, bacteria provide a barrier to the production of vaccines due to their enormous genetic diversity, variety of possible antigens, and capacity to produce a wide range of illnesses with varying results based on the host’s state^5^. Since many bacterial pathogens have diverse protein and polysaccharide antigen architectures due to their genetic variability, developing vaccines is challenging and strain cross-protection is uncommon. In order to increase vaccination coverage and minimize vaccine resistance owing to changes, multicomponent vaccines are often employed for bacteria. Nevertheless, it might be difficult to discover numerous conserved and immunogenic antigens. For example, *K. pneumoniae*, which is a leading cause of sepsis and has rising AMR rates^21^.

Given that there are more than 70 capsular serotypes in the globe, it may not be possible to achieve sufficient strain coverage with a capsule-based vaccination, as has been done with other bacteria like *S. pneumoniae*. For over a century, the Bacillus Calmette-Guerin (BCG) vaccination has been used to prevent *M. tuberculosis* (TB) in young infants. Its efficacy varies (0–80%) in older children and adults^5^. The variable efficacy of tuberculosis (TB) treatments may be attributed to the diverse range of immune responses that children and adults have to a clear illness. Thus, both the host and the pathogen must be considered while developing vaccinations to prevent AMR. Determining the vaccine’s target population and comprehending the protective immune responses present in that group is essential. Furthermore, it is necessary to understand which strains carry the most burden, conduct excellent molecular epidemiological research, and use suitable antigen selection techniques^21^.

mRNA-based nucleic acid vaccines have been under development for more than three decades to create inexpensive, safe, widely applicable vaccinations with minimal side effects. mRNA vaccines, in theory, provide various benefits over traditional vaccines^21–23^. The non-infectious nature of mRNA and its resistance to genome integration confer distinct advantages to mRNA vaccines^24^. mRNA vaccines are similar to DNA vaccines in that they possess the ability to deliver intracellular antigens and overcome the drawbacks of limited immunogenicity and potential non-specific immunity against the vector. Furthermore, they do not pose a threat of DNA integration into the host. mRNA vaccines are more effective, safer, and easier to produce than DNA vaccines^25^. The inflammatory capacity of the mRNA is also diminished by including modified nucleosides. In contrast to conventional vaccines, which induce immunity using attenuated or destroyed viruses or their components, mRNA-based vaccines offer numerous advantages. They are specific in nature, as they solely bind to a particular antigen and elicit a targeted immune reaction including innate immune system, cellular and humoral immune responses^26^. Rapid degradation of mRNA occurs via cellular processes subsequent to the initiation of an immune response. Standardization of mRNA vaccine production is straightforward since alterations in the antigen do not impact the physical-chemical properties of the mRNA backbone. Furthermore, the production process relies on an in-vitro cell-free transcription reaction, which effectively mitigates any safety concerns regarding viral contaminants^27^. Even though the development and manufacturing of mRNA therapy have become safe, capable of programming, adaptable, and economical, the natural characteristics of mRNA and other technological constraints present significant challenges^28^. Immune modulation, upstream processing, duration of expression, stability, requirement for cold chain storage and *in vivo* targeted administration are the three primary constraints (Fig. 1). Researchers are highly intrigued by these challenges, and they are exerting exceptional efforts to discover the corresponding solutions^28,29^.

**Figure 1:**
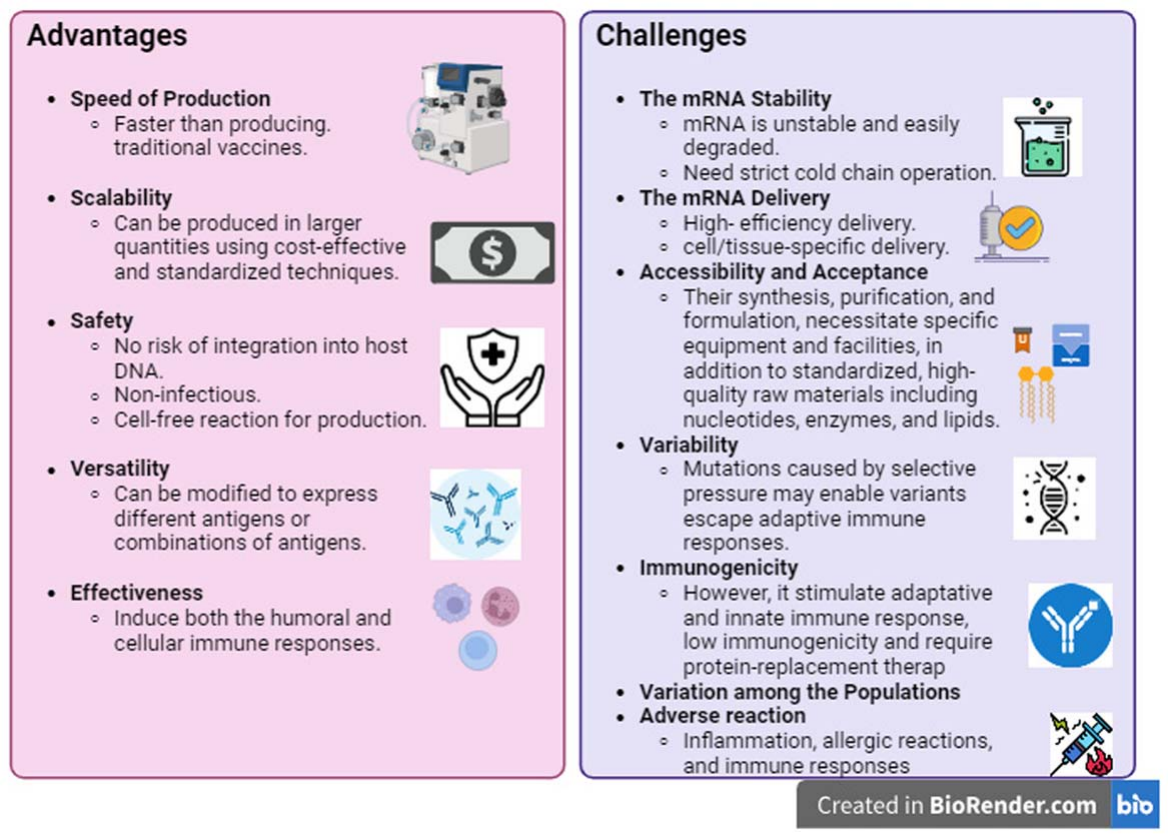
The advantages and challenges of mRNA-based nucleic acid vaccines.

Concerns regarding insertional mutagenesis are unnecessary with mRNA because it doesn’t incorporate into the DNA like certain viral vaccines. mRNA vaccines may be synthesized in a cell-free system, which facilitates quick, scalable, and inexpensive manufacturing^29,30^. Self-amplifying mRNA^23^ and non-replicating vaccines are two types of RNA-based vaccines that employ the host cell’s translational machinery to generate the desired antigens^10^. The non-replicating mRNA vaccine is a simple method in which the injected mRNA is immediately translated into immunogenic proteins in the cytoplasm of transfected cells. Antigen expression caused by a non-replicating mRNA vaccination correlates with the number of transfected cells, hence a high quantity of mRNA must be injected^31^. For transgenic expression, many vector systems have been employed, including replication-deficient viral particles, replication-competent viral particles, and DNA-launched-mRNA vector techniques^32,33^. The engineered DNA-launched-mRNA vectors combined with non-structural proteins (nsp1-4) generate a replicase complex that induces effective transgene expression via a self-amplifying technique^31^. The SAM-based vaccines are made up of an engineered and modified RNA viral genome that encodes nsps, where the structural protein genes are substituted with the targeted specific gene^10^. Therefore, mRNA technologies may provide a fast and significant approach that could be further utilized for vaccines towards AMR infections^10^.

Recently, it was discovered that a SAM vaccine encoding for the backbone protein of pilus island 2a (BP-2a) and a double-mutant of Streptolysin-O (SLOdm) from Group B (GBS, *Streptococcus agalactiae*) and Group A (GAS, *Streptococcus pyogenes*) was immune response-stimulating in mice, provoking both cellular and humoral responses^34^. The antibodies produced by SAM vaccinations provided reliable protection in mouse models against GAS and GBS infections. The immunogenicity of SAM vectors linked to cationic nano-emulsion (CNE) was investigated in CD-1 mice. The geometric mean titres (GMT) of BP-2a after 2 and 3 weeks were 4.7×10^2^ and 4.7×10^3^, respectively, for immunogenicity. Whereas the SLOdm’s GMT after 2 and 3 weeks were 1.25×10^4^ and 2.5×10^4^, respectively^35^.

The saRNA may stimulate immunity against multiple antigens, is affordable and adaptable, and can express protein for up to 60 days, making it an effective chlamydia vaccine platform. A previous study optimized cationic adjuvant formulations (CAFs) for saRNA transport *in vivo* and examined the immunogenicity profile for both humoral and cellular immunity against *Chlamydia trachomatis*’s Major outer membrane protein (MOMP). MOMP-encoding saRNA combined with CAFs produced both MOMP-specific humoral and cellular immunity. Incorporating toll-like receptors (TLR) agonist R848 slightly increased IFN-J+ T-cell responses, but RNA’s self-adjuvating effects dominated the immune response. These studies show that CAFs deliver saRNA efficiently for in-vivo immunogenicity and in-vitro transfections, generating balanced humoral and cellular reactions to protein expression.

Recent research on mRNA vaccine development has focused on viral infections and cancer despite encouraging preclinical results from the malaria vaccine^36^. Given that fungi and bacteria make up the vast majority of AMR pathogens of interest, further research is required to establish the possible application of AMR vaccines^36^. The possible efficacy of an mRNA vaccine toward *Mycobacterium tuberculosis* was investigated by Xue *et al*.^37^ in 2004, who discovered that RNA structures expressing the *M. tuberculosis* MPT83 antigen stimulate the specific T cell and humoral immune responses and provide relatively small but substantial temporary immunity against M. tuberculosis H37Rv infection in mice. Four weeks following the vaccination, the lung bacterial load in the vaccinated mice was roughly one log smaller than in control RNA-vaccinated animals but about 10 folders greater than in BCG-vaccinated mice. In contrast to nowadays, the mRNA platform was far less developed in 2004^37^. In 2010, Lorenzi and colleagues developed a messenger RNA vaccine utilizing the Hsp65 protein derived from Mycobacterium leprae. They demonstrated that administering one dose of 10 μg of naked mRNA-Hsp65 via intranasal route to mice protected them from future problems with a virulent strain of *Mycobacterium tuberculosis*. Furthermore, it was demonstrated that this vaccination induced the specific generation of TNF-alpha and IL-10 in the spleen. The results of these experiments indicated that the populations of CD11b^+^, CD11c^+^, and CD19^+^ cells could acquire the mRNA from 30 minutes to 8 hours. Additionally, mRNA-Hsp65 *in vitro* was found to stimulate the formation of nitric oxide^37^ via TLR7^38^.

Studies indicate that 20–30% of listeriosis cases are fatal, attributing this to the extremely virulent nature of *L. monocytogenes*. Despite the significant mortality rate and escalating antibiotic resistance associated with *Listeria monocytogenes*, there is presently no vaccine that has received clinical approval. Although attenuated *Listeria* strains provide immunity and are being evaluated as vectors for antitumor vaccines, they would be more advantageous in terms of understanding immunodominant vector antigens. Attempts are underway to develop mRNA vaccines for *L. monocytogenes*. A set of 68 Listeria immunopeptides derived from various bacterial surface proteins were recently identified by Mayer and colleagues. These immunopeptides were utilized in the formulation of mRNA vaccines based on lipid nanoparticles and could potentially function as novel antigens. Nucleoside-modified mRNA with α-galactosylceramide (α-GC) as an adjuvant was used to immunize C57BL/6J mice with seven protein antigenic sequences. Several cell lines’ peptide epitopes were derived from the same bacterial surface proteins, which renders them potential vaccine candidates. Specific CD8+ T-cell responses, activation of invariant natural killer T (iNKT) cells, and specific T-cell responses were observed in mice immunized with these highly presented antigens in lipid nanoparticle mRNA vaccines. These findings enable the development of a clinical mRNA Listeria vaccine and improve attenuated Listeria vaccines and vectors, demonstrating immunopeptidomics ability to develop next-generation bacterial vaccines.

Recently, the Israel Institute for Biological Research (IIBR) and Tel Aviv University in Israel, have developed the world’s first mRNA vaccine for bacteria. This study outlined the development of multiple mRNA Lipid Nanoparticle (mRNA-LNP) vaccines targeting *yersinia pestis*, utilizing the F1 capsular antigen. The mRNA-LNP vaccines containing the F1 antigen did not incorporate signal sequences or were connected to human Fc, resulting in significant cellular and humoral immune reactions^39^. Following being tested on mice, it was discovered to be 100% efficient towards Y*. pestis*, the bacteria that was responsible for the horrific Black Plague that occurred from 1347 to 1351^39,40^. According to the researchers, this novel approach will allow for the quick production of efficient vaccines for bacterial infections, particularly those caused by MDR bacteria, in the event of another global new-growing pandemic. Furthermore, one dosage of the vaccine was detected with complete protection only two weeks post-vaccine injection. The outcome was a strong immunological response, with the immune system recognizing the proteins contained in the vaccine as immune-stimulating bacterial proteins^40^. These findings highlight the promise of mRNA vaccines in the fight against AMR bacteria. However, thermostability and the necessity for storage and ultra-cold chain are considered as the main obstacles in the establishment and production of mRNA vaccines against AMR bacteria. All approved mRNA vaccines currently target viral diseases, and there has been no attempt to mix several antigens into one vaccine^41^. It should also be noted that other scientific obstacles in vaccine production, such as the lack of understanding of the factors that induce the human immune response against a certain pathogen, will not be resolved by mRNA technology^41^.

In conclusion, different antibiotic-resistant bacteria contribute to the global problem to different degrees, underlining the need for unique approaches to prevention and management. The economic worth of AMR should be precisely determined since this information is crucial for making decisions. To save these medications for times when they are really needed, more strategies are required to control the use and AMR rates. Implementing infection control and antibiotic stewardship programs to make the best use of currently available antibiotics are examples of non-vaccine alternatives. Although vaccination is the best course of action to address AMR in many aspects, bacteria provide a barrier to the production of vaccines due to their enormous genetic diversity, variety of possible antigens, and capacity to produce a wide range of illnesses with varying results based on the host’s state. However, an extensive number of encouraging safety and effectiveness evidence suggests that mRNA therapies are going to transform the approach of vaccination, particularly against MDR pathogens, cancer immunotherapy, protein replacement therapy, and other areas of contemporary medicine.

## Ethical approval

This article does not require any human/animal subjects to acquire such approval.

## Consent

Informed consent was not required for this editorial article.

## Source of funding

This study received no specific grant from any funding agency in the public, commercial, or not-for-profit sectors.

## Author contribution

M.A.H.A.: conceptualization, data curation, resources, writing—original draft, writing—review and editing; P.C., H.B., N.A. and M.L.A.H.A.: supervision, writing—review and editing; O.P.C.: conceptualization, data curation, supervision, visualization, writing—review and editing. All authors critically reviewed and approved the final version of the manuscript.

## Conflicts of interest disclosure

Not applicable.

## Research registration unique identifying number (UIN)

Not applicable.

## Guarantor

Dr Mai Abdel Haleem Abusalah.

## Availability of data and materials

Data are available upon reasonable request.

## Provenance and peer review

Not applicable.
